# An Integrated Goat Head Detection and Automatic Counting Method Based on Deep Learning

**DOI:** 10.3390/ani12141810

**Published:** 2022-07-15

**Authors:** Yu Zhang, Chengjun Yu, Hui Liu, Xiaoyan Chen, Yujie Lei, Tao Pang, Jie Zhang

**Affiliations:** 1 College of Information Engineering, Sichuan Agricultural University, Ya’an 625000, China; 201902158@stu.sicau.edu.cn (Y.Z.); 202005807@stu.sicau.edu.cn (C.Y.); 202004026@stu.sicau.edu.cn (H.L.); 201902294@stu.sicau.edu.cn (Y.L.); 12340@sicau.edu.cn (J.Z.); 2 Sichuan Key Laboratory of Agricultural Information Engineering, Ya’an 625000, China; 3 College of Mechanical and Electrical Engineering, Sichuan Agricultural University, Ya’an 625000, China; pangtao@sicau.edu.cn

**Keywords:** precision farming, object detection, object tracking, automatic counting, deep learning, computer vision

## Abstract

**Simple Summary:**

To achieve precision and intelligence in farming, automatic recognition and counting of goats are important and fundamental parts of the process of large-scale goat farming. Currently, many farms with low modernization use manual counting, which has the obvious shortcomings of low efficiency and difficulty in avoiding duplication and omissions due to the large population base and frequent counting needs of goats. In order to solve this problem in the farming process, an efficient and accurate goat counting method is urgently needed. In this study, we address the above problem by constructing an integrated deep learning model for automatic detection and counting of goats based on computer vision technology with the Chengdu Ma goat as the research object. It is worth noting that we have improved the model using a series of advanced and effective strategies to enhance the performance of the model. Experiments show that our method can achieve accurate automatic counting of goats in a practical breeding environment. The method is beneficial to the regionalized management of goat barns and can be applied to different goat species with high practicality.

**Abstract:**

Goat farming is one of the pillar industries for sustainable development of national economies in some countries and plays an active role in social and economic development. In order to realize the precision and intelligence of goat breeding, this paper describes an integrated goat detection and counting method based on deep learning. First, we constructed a new dataset of video images of goats for the object tracking task. Then, we took YOLOv5 as the baseline of the object detector and improved it using a series of advanced methods, including: using RandAugment to explore suitable data augmentation strategies in a real goat barn environment, using AF-FPN to improve the network’s ability to represent multi-scale objects, and using the Dynamic Head framework to unify the attention mechanism with the detector’s heads to improve its performance. The improved detector achieved 92.19% mAP, a significant improvement compared to the 84.26% mAP of the original YOLOv5. In addition, we also input the information obtained by the detector into DeepSORT for goat tracking and counting. The average overlap rate of our proposed method is 89.69%, which is significantly higher than the 82.78% of the original combination of YOLOv5 and DeepSORT. In order to avoid double counting as much as possible, goats were counted using the single-line counting based on the results of goat head tracking, which can support practical applications.

## 1. Introduction

China has become a big consumer of mutton. According to the data of The National Bureau of Statistics of China, 330.45 million sheep were sold in 2021, with an increase of 11.04 million and 3.5 percent over the previous year. Mutton output was 5.14 million tons, with an increase of 220,000 tons and the growth rate of 4.4%. At the end of 2021, the total number of sheep in China was 3.19.9 million, with a year-on-year increase of 4.3% [1]. With the increase in the number of sheep breeders, management problems have also emerged. Most of the farms are currently using artificial breeding methods, and most of them use tagging of ears or use channels to count sheep. The former is labor-intensive, inefficient, and requires direct contact with sheep, which may cause different degrees of injury to them; while the latter also has certain limitations: the construction of channels is costly, they are not easy to move, and the universality is not high. Therefore, we need to use modern and intelligent methods to improve the efficiency of sheep counting and promote the development of sheep farming towards precision and automation.

In recent years, there have been many research studies applying deep learning and computer vision techniques to livestock farming to promote the development of livestock intelligence. In 2019, Tian et al. presented a modified counting convolutional neural network model according to the structure of ResNeXt to address the pig counting problem [2]. In 2020, Xu et al. developed an auto sheep counting system based on multi-object detection, tracking, and extrapolation techniques [3]. In 2021, to address problems caused by the partial occlusion, a two-stage center clustering network (CClusnet) was developed to improve automated piglet counting performance [4]. Jensen et al. made use of convolutional neural networks with a linear regression output to realize automatic counting and positioning of slaughter pigs within a pen [5]. In 2022, a semantic segmentation and counting network was proposed to improve the segmentation accuracy and counting efficiency of pigs in complex image segmentation [6]. Using a camera in a hallway, Kim et al. used a deep-learning-based video object detection and tracking method to count the number of pigs passing through the counting zone [7]. Huang et al. proposed an optimized cow tail detection and tracking method based on an improved single shot multibox detector (SSD) and Kalman filter, which provides a new solution to automatic cow detection and tracking in smart livestock farming [8]. To minimize costs when using satellite images of animals, the lowest spatial resolution data that enable accurate livestock detection should be selected. Therefore, Brown et al. determined the association between object detector performance and spatial degradation for cattle, sheep, and dogs [9]. 

A review of related work in recent years reveals that although much excellent work has emerged in the field of detection and automatic counting of livestock, relatively little research has been conducted on detection and automatic counting of sheep, pending the application of the latest advanced technology to solve practical problems in sheep breeding. This paper takes the Chengdu Ma goat, a fine breed in China, as the object of research, with the aim of being able to use computers to detect goats quickly and accurately in a barn and to count them automatically in order to improve the efficiency of goat farm management and to promote goat farming in the direction of modernization, intelligence, and scale.

Different from the methods of realizing livestock counting by direct detection or segmentation, this paper describes a method of counting goats through multi-object tracking. Since goats are constantly moving around the barn, counting by direct detection or segmentation is likely to result in duplicate counting or missed counting due to occlusion. The multi-object tracking algorithm assigns a unique ID to each target, and this ID remains constant throughout the sequence, thus ensuring the accuracy of counting. Tracking by detection is the mainstream approach of multi-object tracking algorithms. Before tracking, the object information in each frame is obtained in advance by the detection algorithm, so the performance of this method depends on the quality of the detection algorithm. Since YOLOv5 is the most notable and convenient one-stage detector, we selected it as our baseline of detector, and used a series of advanced strategies to improve its performance. In order to reduce the overlap rate of detected objects, we only detected the goat heads. Then, we input the information such as bounding box and confidence of the detected goat heads into DeepSORT [10], a well-known multi-object tracking algorithm, to realize the tracking and counting of goats.

## 2. Materials and Methods

### 2.1. Data Acquisition

The data used in this research were collected from Chengdu Ma goat farm of Chengdu Xilingxue Agricultural Development Co., Ltd. (Chengdu, China) For the image data of the goats, we used a Canon EOS 850D to shoot in a variety of ways, including long distance, close up, diagonally downward, and parallel to the ground. We randomly divided the resulting 623 images into a training set and a test set in a ratio of 7:3. For the video data of the goats, each pen in the barn had an EZVIZ C3WI wireless surveillance camera recording at an angle angled downward toward the goat pen. The video data we collected came from a dozen different goat pens on the farm, with the number of goats in each pen varying from a dozen to two dozen.

### 2.2. Data Preprocessing

Data augmentation [11] is an effective method to expand data samples. Deep learning is based on a large amount of data. The larger the scale and higher the quality of the data, the better the generalization ability of the model. However, when collecting data in practice, the data usually cannot contain all conditions, such as different lighting conditions. It is difficult to control the lighting during data image acquisition, so data augmentation with lighting variation needs to be considered when training the model. Data augmentation is achieved by altering or learning from incomplete and small-scale datasets, and the generated data are sufficient and reasonable.

**Figure 1:**
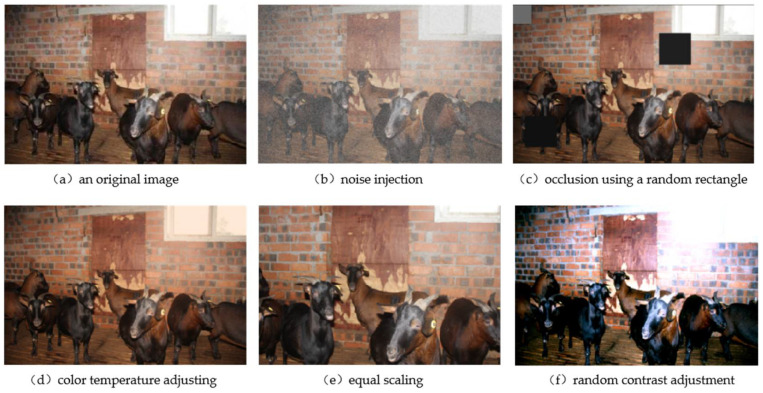
Images of single sample augmentation.

Single sample data augmentation is the most simple and common way to augment data by using geometric changes [12], including flipping, rotation, clipping, scaling deformation, and affine operations. Color change is a data augmentation method of changing the content of the image itself, and its common operations include noise, blur, random erasure, and data augmentation methods of color transformation such as the superpixel method. These methods increase the diversity and variation in data samples to a certain extent. Figure 1 shows some images of single sample augmentation.

**Figure 2:**
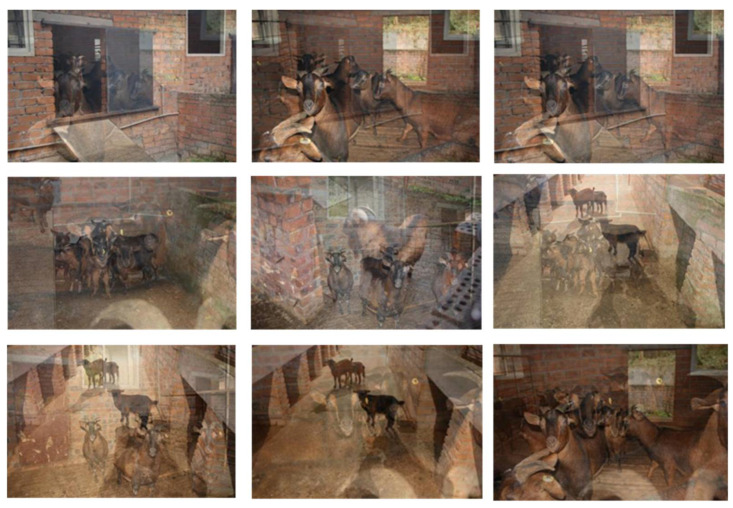
Images after Mixup augmentation processing.

Mixup [13] is an algorithm for mixing and enhancing images in computer vision. It mixes images of different types and linearly understands the training samples by linear interpolation. It can lower the absoluteness of sample judgment, reduce the fitting, build new training samples and labels to achieve the purpose of expansion of training data, and enhance the generalization ability of the model. Figure 2 shows some images after using Mixup.

**Figure 3:**
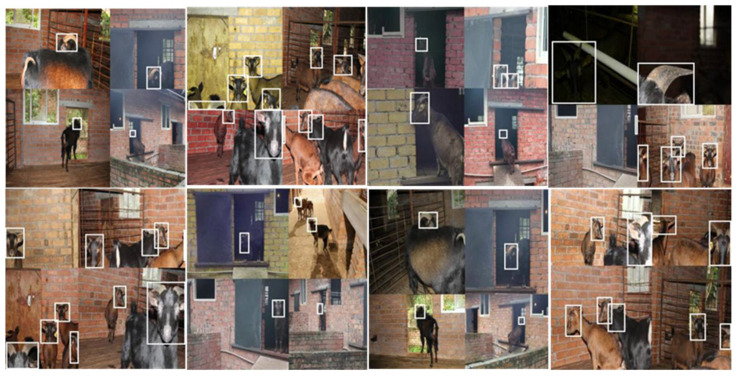
Images after Mosaic augmentation processing.

Mosaic is a data augmentation method from the paper of YOLOv4 [14], which uses four images. The four images are spliced to one image as training data by flipping, changing the color gamut, and scaling. It has the following two main advantages: First, it can enrich the object detection background and small objects, which means expanding the training data and improving the robustness of the model. Second, during batch normalization, the data of four images are calculated at the same time, reducing the dependence on batch size, so that one GPU can achieve better results. Figure 3 shows some images after using Mosaic.

In the experiment, various means are used to enhance the amount of data and improve the generalization ability of the model, so that the model can achieve relatively good results in all kinds of complex situations.

### 2.3. Rand Augment

At present, data augmentation strategies are mainly based on people’s subjective thoughts and relevant experience. Relevant practitioners or professionals can analyze and configure reasonable data augmentation strategies based on possible situations encountered in practical applications. Therefore, it can be seen that designing a good data augmentation method mostly comes from the accumulation of long-term experience, and even if a data augmentation method performs well, it may not perform well in other datasets. Therefore, a reasonable search algorithm is designed to automatically search for the best data augmentation strategy for the dataset. At the same time, the idea of RandAugment [15] is used to ensure the diversity of data augmentation strategies and reduce the search space.

By directly adopting a parameterless process to reduce the search space, and then selecting a data augmentation function with a probability of 1/K, RandAugment can provide $K^{N}$ transformable strategy for the actual task if there are N data augmentation methods available. An intensity range of 0 to 10 (integers only) is then set for each data augmentation function. In order to further reduce the space needed for searching, a schedule is used to indicate that the intensity of each method varies with the iteration process. Four selection strategies were explored, and fixed intensity, random selection intensity, intensity that increases linearly with the number of iterations, and randomly selected intensity with upper bounds were used throughout the training. By using the RandAugment method, the augmentation methods mentioned above can be reasonably selected, so that the final model can obtain good accuracy in the actual goat barn environment.

### 2.4. Object Detection

Object detection is to scan and search in images and videos to find out all objects of interest and determine their categories and locations. Object detection combines object recognition and localization to achieve real-time and accurate object detection in a specific environment. It is widely used in various aspects such as intelligent traffic, pedestrian counting, animal recognition, agricultural product pest identification, defect detection, and so on. Convolutional neural network [16] (CNN) is widely used as an important part of computer vision, and object detection technology based on deep learning has attracted much attention. Among the detection algorithms that pursue both accuracy and speed, the one-stage object detection algorithm based on deep learning stands out, which has a simple structure and integrates feature extraction, object classification, and position regression into one stage. Compared with other object detection algorithms, it is more suitable for real-time detection tasks with higher efficiency. A common choice is YOLO (You Only Look Once), which treats detection as a regression problem and possesses an extremely fast speed.

YOLO series algorithms are improving in speed and accuracy. YOLOv1 [17] uses predefined candidate areas to divide the input image into grids, and each grid can predict multiple bounding boxes. Finally, only one bounding box with the highest IoU is selected as the output of object detection. When there are multiple objects in the grid, only one object can be predicted, which is not effective for dense and small objects. YOLOv2 [18] adopted many improvement measures for YOLOv1, such as normalization of each layer of the network and use of a high-resolution classifier, which improved the mAP of the model, but failed to solve the problem of small object recognition. YOLOv3 [19] introduced residual modules to deepen the network structure. Multi-scale feature information can predict different sizes of objects and improve the accuracy of small object detection. YOLOv4 [14] and YOLOv5 have further improved the accuracy and speed. Compared with other versions of YOLO, YOLOv5 has the advantages of flexibility and light weight [20].

### 2.5. Our Improved YOLOV5-Based Detection Network for Chengdu Ma Goat

#### 2.5.1. AF-FPN

**Figure 4:**
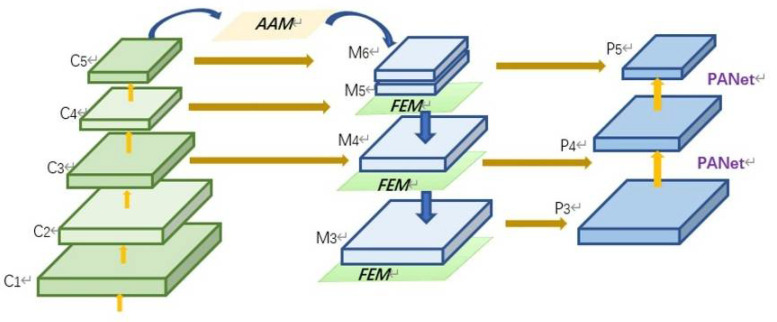
The structure of the AF-FPN [22].

When detecting goats, due to the movement of individuals, the distance between the goat and the shooting device varies greatly, so there are differences in the size of different individuals, which can cause certain influence on the accuracy of the detection. The feature pyramid networks [21] is widely used to solve this kind of problem, but it is very difficult to improve the precision of multi-scale detection while ensuring the real-time detection in practical applications. AF-FPN [22] adds the adaptive attention module (AAM) and feature enhancement module (FEM) successively on the basis of the traditional feature pyramid network in order to reduce the loss of context information of high-level feature maps, which improves the accuracy and inference speed of the model at the same time. The structure of AF-FPN is shown in the Figure 4.

**Figure 5:**
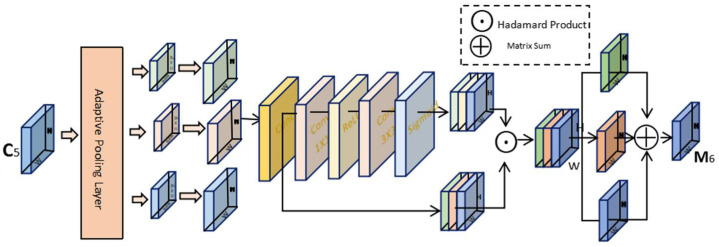
The structure of the AAM [22].

C5 is the input of the AAM, which is obtained from the input image through convolution. Firstly, the different scales of context features are obtained under the function of the adaptive pooling layer. Then, the 1×1 convolution is used to obtain the same channel dimension and bilinear interpolation is used to upsample them to the scale of S, laying the foundation for subsequent fusion. The spatial attention mechanism merges the channels of the three context features and generates corresponding spatial weights for each feature map. The generated weight map and the feature map after merging channels are subjected to the Hadamard product operation, which is separated and added to the input feature map M5 to aggregate context features into M6. The final feature maps have rich multi-scale context information, which to a certain extent alleviate the loss of information due to the reduction in the number of channels [22]. The structure of AAM is shown in Figure 5.

**Figure 6:**
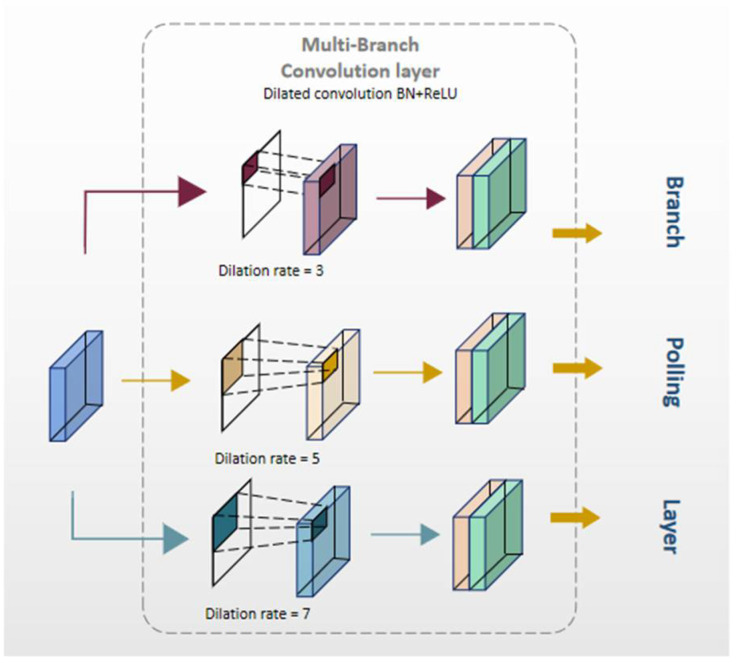
The structure of the FEM [22].

FEM mainly uses dilated convolution to adaptively learn different receptive fields in each feature map according to different scales of detected image features, so as to improve the accuracy of multi-scale object detection. It can be divided into two parts: multi-branch convolution layer and multi-branch pooling layer. The multi-branch convolutional layer is used to provide input feature maps with receptive fields of different sizes through dilated convolution, and the average pooling layer is used to fuse image information from the three receptive fields. The structure of FEM is shown in the Figure 6.

#### 2.5.2. Dynamic Head

In the detection task, we generally focus on the three dimensions of scale, spatial, and task to continuously improve the performance of the model. The Microsoft Research Institute has proposed a dynamic detection head architecture, Dynamic Head [23]. Through the attention mechanism, three different forms of object detection methods are unified into one, which can greatly improve the representation ability of the detection head.

Direct attention on the input tensor of the detector’s head part is an intuitive scheme, but this approach is difficult to optimize and very computationally intensive. Thus, the paper [23] proposes a separated attention mechanism to perform attention separately for each independent dimension in feature maps, which is described as follows:

1. Scale-aware attention (level-wise) $\pi_{L}$: Different levels of feature maps correspond to different object scales, so increasing attention level-wise enhances the scale awareness of the object detector.
2. Spatial-aware attention (spatial-wise) $\pi_{S}$: Different spatial locations correspond to geometric transformations of the object, so adding attention spatial-wise enhances the spatial location perception of the object detector.
3. Task-aware attention (channel-wise) $\pi_{C}$: Different channels correspond to different tasks, so adding attention to the channel dimension can enhance the perception of object detector for different tasks.

**Figure 7:**
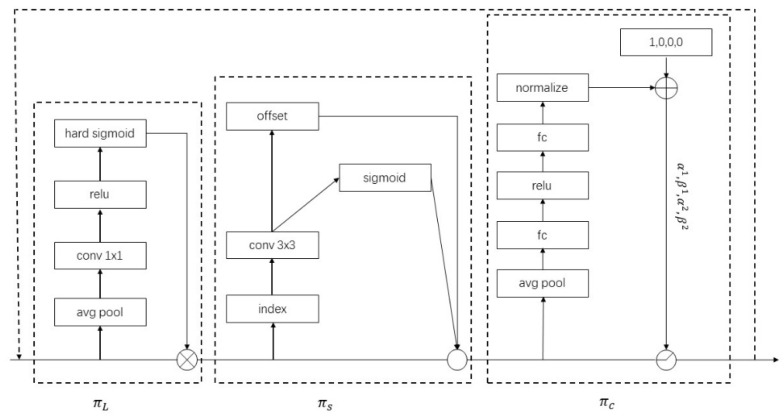
The structure diagram of the Dynamic Head block [23].

Dynamic Head is the unification of these three types of attention into one efficient attention learning problem. The structure of the Dynamic Head block is shown in Figure 7.

**Figure 8:**
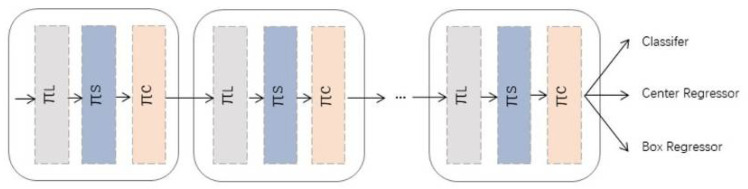
An example of applying Dynamic Head blocks to a one-stage object detector [23].

As a universal detector block, Dynamic Head (DyHead) can be well integrated into all kinds of object detection structures. In this study, YOLOv5 is adopted as the one-stage object detection model, and DyHead blocks are incorporated into the model as shown in Figure 8. Because of the advantages of multiple attention mechanisms, it can handle multiple tasks simultaneously. In this way, the architecture of the object detection model can be further simplified, and the efficiency can be improved.

### 2.6. Object Tracking

Object tracking is based on the information before and after the video data to roughly estimate the location and state of the object to be detected in the scene, such as it can roughly be divided into single object tracking (SOT) [24] and multiple object tracking (MOT) [25] level. In this paper, we mainly discuss multiple object tracking. As one of the main tasks in the field of computer vision, its core aim is to identify and track objects belonging to one or more categories, such as pedestrians on the road, cars in motion, and animals in the natural environment, without any prior knowledge about the number of objects and external representations [26]. The difference from the object detection algorithm discussed above lies in that its output is a set, which is composed of rectangular boundary boxes identified by coordinate information, height, and width. The MOT algorithm can also associate the individual object with each boundary box. In this way, objects within the class can be effectively distinguished. Multi-objective tracking has attracted more and more attention because of its great potential in practical life. As a mid-stream task, it can serve more complex downstream tasks. How to explore the rational application of multi-objective tracking technology in animal husbandry still needs to be further explored.

Under the actual conditions of the goat shed, because the appearance of goat objects to be detected may change, and very serious shelter problems may occur under different environmental conditions, how to use multi-object tracking algorithm to realize automatic counting of goats is one of the efficient methods to get rid of high labor costs and low statistical efficiency.

### 2.7. DeepSORT-Based MOT Network for Automatic Countering

DeepSORT [10] is an improved version of the SORT [27] multi-object tracking algorithm, designed with a new association method that improves the accuracy of tracking objects that are occluded for long periods of time and reduces the frequent switching of ID. Because of the obvious problem of frequent switching of the ID of the SORT algorithm, it is only suitable for objects with few occlusions and relatively stable motion. DeepSORT implements association metrics by combining more accurate metrics of action and appearance information, using CNN to extract features that increase robustness to missing and occlusion, while being easy to implement, efficient, and also suitable for online scenarios.

The DeepSORT algorithm can be divided into four core parts: trajectory processing and state estimation, correlation measurement, cascade matching, and depth feature descriptor. The algorithm of trajectory processing and state estimation is similar to the SORT algorithm, which is mainly divided into motion state estimation, and object creation and removal. The DeepSORT algorithm uses eight parameters (u, v, gama, h, x1, y1, gama1, h1) to describes the object motion state. Among these parameters, (u, v) are the central coordinates of the object anchor box, gama is the aspect ratio of the object anchor box, h represents the height of the object anchor box, and the other four variables are the motion information of the object corresponding to the image coordinates. A standard Kalman filter based on a constant velocity model and linear observation model is used to predict the motion state of the object, and the predicted result is (u, v, gama, h). Set a threshold $A_{max}$, and set a counter $a_{k}$ to represent the number of frames between the last appearance of the kth tracking object and the current frame. Every time the object appears, the counter will be refreshed, that is, the counter will be set to 0. If $a_{k}$ > $A_{max}$, the object k tracking ends. If there is an object that can never be matched with an existing path during the object matching process, it is considered that the object may be a newly emerged object. If the object is detected in the next 3 frames consecutively, it is recognized as the emerging tracking target and a new tracking path is generated with this object as the starting object, otherwise no new tracking path is generated.

**Figure 9:**
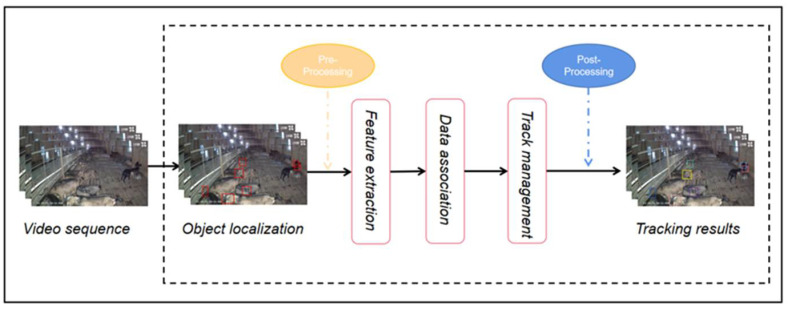
The flow chart of goat tracking.

The DeepSORT tracking algorithm contains six main steps. The first step is object localization, i.e., detection: in the context of DeepSORT, this step is equivalent to a Kalman filter sensor. The second step is preprocessing: NMS (Non-maximum Suppression) and confidence threshold detection (i.e., discarding detections whose confidence is less than the threshold). The third step is feature extraction: in the context of DeepSORT, this part is the extraction of appearance features by the re-ID model. The fourth step is data association: in the context of DeepSORT, that is, the current kth frame of detection is matched and the track prediction generated in the Kalman filter prediction stage according to the previous frame. In the context of DeepSORT, it is cascade matching and IoU matching. The fifth step is track management, including track update in Kalman filter, and initialization and deletion of track. After the last postprocessing step, the tracking results are obtained. Figure 9 shows the flow of goat tracking.

The head of the goat is detected by our improved YOLOv5 network, and then the extracted head area information is sent to DeepSORT. The DeepSORT detector first initializes, then identifies the goat based on the incoming goat head information and updates the positions of the already tracked targets, and finally obtains the bounding boxes with ID to complete the tracking of the goat.

**Figure 10:**
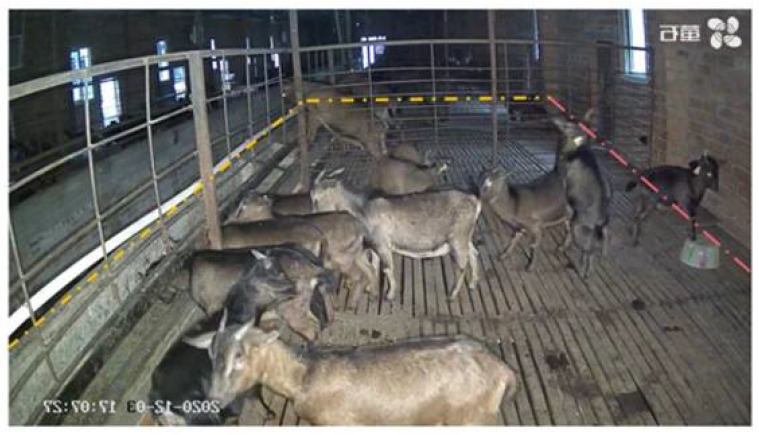
The diagram of counting area.

**Figure 11:**
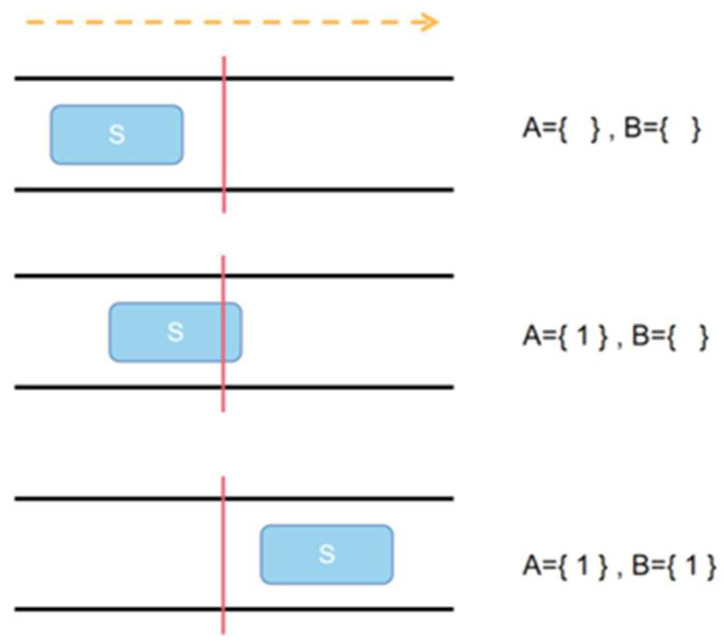
The schematic diagram of single-line counting method.

In order to avoid repeated counting or missing counting as much as possible, goats are counted using the single-line counting [28] based on the results of goat head tracking. For automatic counting of goats, it is necessary to delineate the corresponding counting area for the goat barn, as shown in Figure 10. The yellow line and the red line together constitute the automatic counting area. The red line is the core of the single-line counting, and the result of the goat head tracking is used to determine whether the goat before and after the red virtual line is the same goat. When the center point of the tracking target box touches the red line, the counting starts, and when the center point leaves the red line, the counting ends. First, we set up the sets A and B, where A records the set of all target IDs that have intersected the counting line, and B records the set of all target IDs that have intersected the counting line and then left the line. Figure 11 shows the states in the two sets when the target with ID 1 is tracked by DeepSORT from left to right across the counting line (indicated by the red line in the figure) for three consecutive moments.

State A: A target appears in the field of view from left to right, assuming that the tracker has set its ID to “S”. At this point there are no elements in either set A or B.

State B: The target “S” continues to move to the right until it intersects the line, at which point the ID “1” is added to the set A. At this point of intersection, the target does not leave the line, so the set B still does not contain any elements.

State C: The target “S” continues to move to the right until the moment when the whole is detached from the line, when the counter finds that the target “S” is in the set A and has left the counting line, then the ID “1” is added to the set B.

Eventually, after all targets have gone through the above process of target “S”, the number of all elements in set B is the number of counts.

### 2.8. Evaluation Methods

#### 2.8.1. Precision

Although the confusion matrix can objectively evaluate the prediction performance of the model, in the object detection task, the operation of the confusion matrix is not intuitive. In this paper, the accuracy rate is introduced based on the confusion matrix, that is, the ratio of the correct matching with the goat labeling frame is traversed through all the currently detected prediction frames. The calculation formula is:

(1) $$precision=\frac{TP}{TP+FP}$$

#### 2.8.2. Recall

Recall rate is the percentage of all positive samples in the test set that are correctly detected as positive samples, that is, the ratio of prediction box to labeling box of all goats in the test set of this study, and the calculation formula is:

(2) $$recall=\frac{TP}{TP+FN}$$

Under the same accuracy, the higher the recall rate, the better the performance of the model.

#### 2.8.3. mAP

When the IoU threshold is constantly changed, the accuracy and recall rate will constantly change, resulting in the precision–recall curve. When judging the performance of a model, it is often judged by whether the increase in recall rate will cost the loss of too much precision rate. With the increase in recall rate, the precision rate of a model with excellent performance always remains high. The mean average precision (mAP) is an average value, which is calculated by detecting the average precision (AP) values of multiple objects in a task for an average object. The value of AP is the area of a P-R curve accurately drawn by predicting the precision and recall of the experimental results obtained from the analysis.

(3) $$mAP=\frac{\sum_{n}precision}{n}$$

#### 2.8.4. Average Overlap Rate

Overlap rate is used to judge and predict the degree of overlap between rectangular frames and labeled rectangular frames: the higher the value, the higher the degree of overlap, that is, the closer the two frames are to each other, the higher the object tracking accuracy. Overlap rate is completely consistent with the definition of IoU index discussed above, but it is mainly used in the field of object detection, so it is described by overlap rate in object tracking tasks. The calculation formula of overlap rate is as follows:

(4) $$OR=\frac{S_{d}\cap^{\;}S_{t}}{S_{d}\cup^{\;}S_{t}}$$

The calculation formula of the average overlap rate is as follows:

(5) $$AOR=\frac{\sum_{n=1}^{N}OR_{n}}{N}$$

$S_{d}\;$ represents the predicted bounding box of the object, $S_{t}$ represents the labeled bounding box, and N represents the total number of objects detected in the total region.

#### 2.8.5. Mean Center Position Error

Center location error is used to describe the accuracy of the tracking object of the model at a certain time. The smaller the center location error is, the higher the accuracy of object tracking will be.

(6) $$CLE=\sqrt{{\left(h_{e}-h_{f}\right)}^{2}+{\left(y_{e}-y_{f}\right)}^{2}}$$

The calculation formula of the mean center position error is as follows:

(7) $$ACLE=\frac{\sum_{n=1}^{N}CLE_{n}}{N}$$

where $h_{e},y_{e}$ represent the central point coordinates of the predicted bounding box of the object, respectively, and $h_{f},y_{f}$ represent the central point coordinates of the labeled bounding box, respectively.

## 3. Results

### 3.1. Experimental Effect Analysis of Object Detection Model

**Figure 12:**
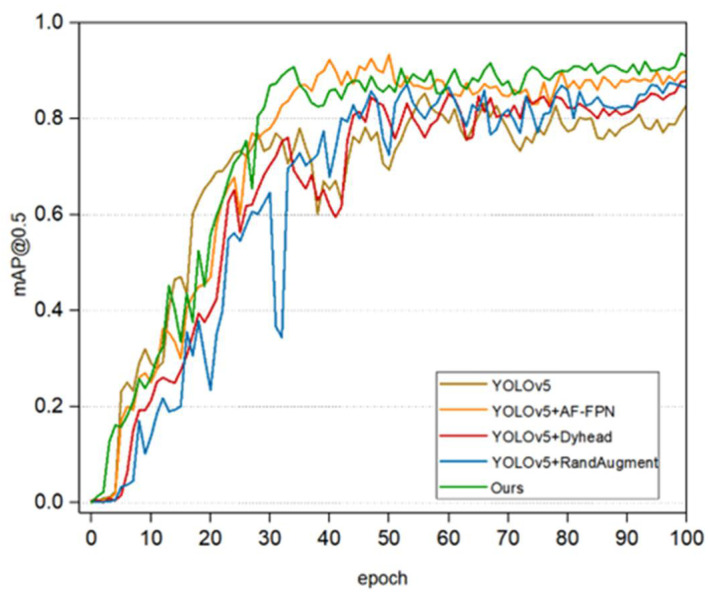
Changes in mAP@0.5 during training.

Effective detection of goat heads is an important basis for goat counting. The practical utility of the improved model is verified by adding different blocks to YOLOv5 separately. Figure 12 shows the changes in mAP during training.

The RandAugment strategy is used to solve the root cause of data augmentation problems, which can be used as an external tool with good results when acting on this network. Table 1 shows the ablation study on data augmentation methods.

By replacing the original feature pyramid network in YOLOv5 with AF-FPN, the detection performance of the YOLOv5 network for multi-scale objects is improved.

DyHead is incorporated to the detection head part, which integrates object detection heads and attention mechanisms. The DyHead structure can be configured flexibly. In the detection model for the head area of the goats, four DyHead blocks are configured, and the order of the attention mechanisms is adjusted in modules 2 and 4 by placing the spatial-aware attention module in front and the scale-aware attention module in the back to dynamically adapt to different conditions of the goat population. The experimental feedback shows that the mAP is improved to a certain extent compared to the sequential stacking, while basically not increasing the additional computation. Table 2 shows the ablation study on our improvements.

### 3.2. Experimental Effect Analysis of Object Tracking and Automatic Counting Model

**Figure 13:**
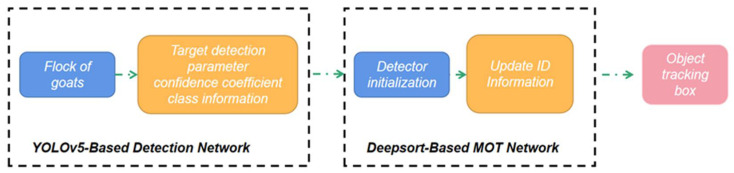
The combination of object detection network and object tracking network.

**Figure 14:**
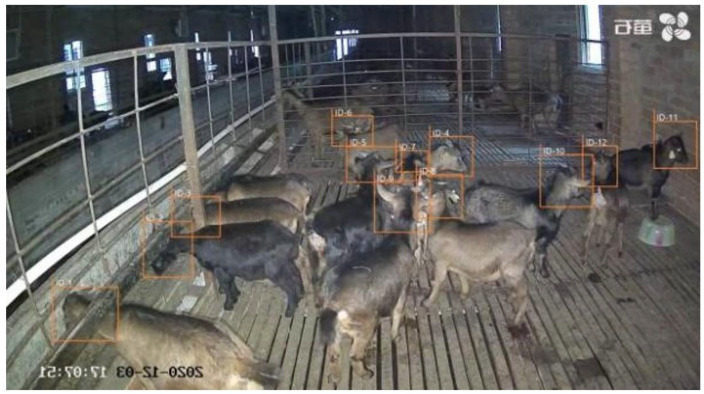
The results of goat tracking.

The goat counting model proposed in this paper has a higher tracking success rate than the original YOLOv5 + DeepSORT model, and is more effective and stable in goat counting. Due to the stronger detection performance of the improved YOLOv5, it is able to capture the goat head information better, making the overall tracking performance able to meet the normal application requirements. Figure 13 shows the combination of object detection network and object tracking network. Table 3 shows the comparison between the original YOLOv5 + DeepSORT and our method. Figure 14 shows the results of goat tracking.

We manually counted the number of goats in the counting area in the video frames and compared them with the counting results of the model. To reflect the good counting performance in each time period, the counting results were counted in 15 s intervals, and the experimental results are shown in Table 4. It is found that when the counting is first performed, the model is able to obtain a count value that is basically consistent with the actual value because the number of goats entering the counting area is small. With the passage of time, the number of goats entering the counting area gradually increased, leading to an increase in the frequency of overlap and occlusion. When the count reaches more than 20 goats, the problem of missed detection easily occurs. Therefore, reasonable control of the number of goats entering the counting area can make our method work well in a practical environment.

We tested the counting accuracy of several recently proposed animal counting methods with our dataset, and the results are shown in Table 5, where the counting accuracy shown in the table is the average of the counting accuracy of three goat pens. The improved SSD [29] is a detection-based animal counting method that is mainly used for goat detection and counting of static top-view images of grassland goats captured by drones. The LA-DeepLab V3+ [6] is originally proposed to solve the problem of segmentation and counting of pigs, and the method achieves good results in counting pigs due to their poor mobility. However, the detection and segmentation-based counting methods are not as effective as the tracking-based methods for counting Chengdu Ma goats in the goat pen environment due to the good mobility of Chengdu Ma goats.

## 4. Discussion

In this paper, an integrated goat head detection and automatic counting method based on deep learning is discussed. We acquired video data from surveillance cameras deployed in the goat barn and used the improved YOLOv5 algorithm to detect the head area of Chengdu Ma goats, and then combined it with the DeepSORT tracking algorithm to ensure that the same goat within the video was not counted repeatedly. Combined with the setting of counting area and single-line counting method, it achieved efficient automatic counting.

Since each goat pen in the goat barn is equipped with only one camera and the location of the camera is fixed, the video is captured from a single angle and the information obtained about the counting area is limited. In the areas where goats are densely distributed in goat pens, occlusion is a serious problem, which affects the counting results of the model. Therefore, to realize more accurate detection and automatic counting, farms should consider deploying cameras directly above each goat pen or deploying multiple cameras for each goat pen to collect information from all directions and multiple angles in the farming environment. This can integrate information from multiple angles to assist in accomplishing accurate identification of individual goats and counting of goats, which is a good solution to the occlusion problem.

In terms of inference speed, the one-stage object detection model YOLOv5 is selected for improvement in order to meet the demand for real-time processing of the video data captured by the camera. Compared with the two-stage algorithm, the one-stage object detection model has a higher inference speed under the same hardware conditions.

Sarwar et al. [30] combined the deep learning algorithm and Unmanned Aerial Vehicle (UAV) technology and used the convolution neural network to count sheep through video streaming collected by drones under different climate conditions. However, the vertical angle image acquisition is mainly used in a free-range situation, so it cannot be extended to the situation of the goat barn. Shao et al. [31] proposed a cattle detection and counting system based on the convolutional neural network in order to help the management of grazing cattle. The system uses aerial images taken by UAV. For species such as goats, which are mainly active in mountainous and hilly areas, it is difficult to obtain UAV images because they are susceptible to the influence of covered vegetation complex terrain. Zhang et al. [28] designed a channel for sheep entering and leaving, collected corresponding video images at the exit position, constructed a counting model, and deployed it in the embedded AI equipment to realize sheep counting, which has good application only in large farms with modern facilities.

Different from the aforementioned studies, we conduct a study for goats. We constructed a video dataset of Chengdu Ma goats that can be used for object tracking and integrated the improved object detection algorithm and the object tracking algorithm into a framework that can effectively solve the counting problem of captive goats without additional infrastructure assistance, which has good applicability in a practical application scenario. We believe that in the near future, combined with our work and that of other authors [32], intelligent breeding and management of Chengdu Ma goats will become possible.

Based on the above discussion, we believe that our proposed method is an effective exploration and discovery of the development of the intellectualization of animal husbandry.

## 5. Conclusions

In this study, deep learning technology is applied to the goat breeding process, and an integrated framework is proposed to solve the automatic counting problem of goats. Firstly, the goat video image dataset for object tracking was constructed. RandAugment was used to reduce the parameter space of data augmentation, and the appropriate augmentation method under the actual goat barn condition was explored, which can be bundled with the model training process completely. In addition, the AF-FPN and Dynamic Head blocks were fused into the improved YOLOv5 model. After improving, the mAP value of 92.19% was obtained, which makes the model effectively capture the information of goat heads. The information obtained in the detection process was passed into the DeepSORT as auxiliary information. For low precision in the case of object occlusion, the DeepSORT tracking algorithm uses cascade matching to enable the ID of the occluded objects to be re-matched. The average overlap rate of our proposed method reached 89.69%, which is significantly improved compared with the original YOLOv5 combined with DeepSORT. Our experiment shows that applying the tracking results to the single-line counting method allows for effective counting of goats. Therefore, we have reason to believe that the automatic goat counting method described in this paper meets the requirements of practical application. It has certain value in the practical application of the goat farming industry and also provides a new idea for related intelligent animal husbandry production.

## Figures and Tables

**Table 1:** Ablation study on data augmentation methods.

Methods	mAP@0.5 (%)
YOLOv5	82.97
YOLOv5 + Mixup	83.32
YOLOv5 + Mosaic	83.87
YOLOv5 + Mixup + Mosaic	84.26
YOLOv5 + RandAugment	85.13
YOLOv5 + Mixup + Mosaic + RandAugment	85.35

**Table 2:** Ablation study on our improvements. All of the following methods use Mixup and Mosaic by default.

Methods	mAP@0.5 (%)	Inference Time (ms)
YOLOv5	84.26	23.3
YOLOv5 + AF-FPN	86.62	22.4
YOLOv5 + DyHead	88.21	26.7
YOLOv5 + RandAugment	85.13	23.7
YOLOv5 + AF-FPN + DyHead + RandAugment	92.19	25.6

**Table 3:** Comparison of object tracking algorithms.

Methods	Average Overlap Rate (%)	Mean Center Position Error
YOLOv5 + DeepSORT	82.78	8.56
Ours	89.69	5.92

**Table 4:** Comparison between quantity in reality and quantity calculated by the model.

The Time Interval	1~15 s	16~30 s	31~45 s	46~60 s
Quantity in reality/goat	7	11	17	26
Quantity calculated by the model/goat	7	11	17	25

**Table 5:** Comparison of several animal counting methods.

Methods	Counting Accuracy (%)
Improved SSD [29] (detection-based)	90.38
LA-DeepLab V3+ [6] (segmentation-based)	94.23
YOLOv5 + DeepSORT (tracking-based)	96.15
Ours (tracking-based)	98.08

## Data Availability

The data used to support the findings of this study are available from the corresponding author upon request.
